# Unraveling Intractable Headache in Acute Myeloid Leukemia With Coexisting Migraines and Chiari Malformation

**DOI:** 10.7759/cureus.95259

**Published:** 2025-10-23

**Authors:** Laveena Singla, Hannah W Austin

**Affiliations:** 1 Neurology/Epilepsy, University of Mississippi Medical Center, Jackson, USA; 2 Neurology, University of Mississippi Medical Center, Jackson, USA

**Keywords:** acute myelogenous leukemia, aml mutation, case report, cns leukemia, headache

## Abstract

The role of acute myelogenous leukemia (AML) in affecting the central nervous system (CNS) is not well-defined, with reported incidence rates varying widely. Central nervous system involvement is typically regarded as rare during the initial stages of AML. We present a case of a 28-year-old woman who had AML with CNS infiltration that manifested as a complex presentation of persistent headaches in the context of multiple confounding factors, including a history of intractable migraines, recent discontinuation of Erenumab, elevated risk of idiopathic intracranial hypertension due to a body mass index of 45, and worsening herniation of a Chiari I malformation. Notably, the patient lacked the typical imaging features usually associated with CNS leukemia. This case highlights the need for proactive assessment of CNS involvement in AML patients who carry high-risk mutations and present with neurological symptoms. It further highlights the importance of having a high index of suspicion for CNS disease in AML patients presenting with headaches, even in the absence of classical radiographic findings of leptomeningeal spread.

## Introduction

Central nervous system (CNS) infiltration by acute myelogenous leukemia (AML) is an uncommon occurrence, with reported incidence rates ranging from 1.11% in newly diagnosed AML cases to as high as 33% in cases of relapsed AML [1,2]. Specific high-risk mutations in AML, including FMS-like tyrosine kinase 3 (FLT3)-Internal Tandem Protein (ITD), abnormalities in chromosome 11, and inv(16) cytogenetic changes, are linked to a higher likelihood of CNS involvement [3]. When assessing CNS imaging, key findings that are highly indicative of CNS leukemia involvement include pachymeningeal enhancement, leptomeningeal enhancement, and enhancement of cranial nerves.

## Case presentation

**Figure 1:**
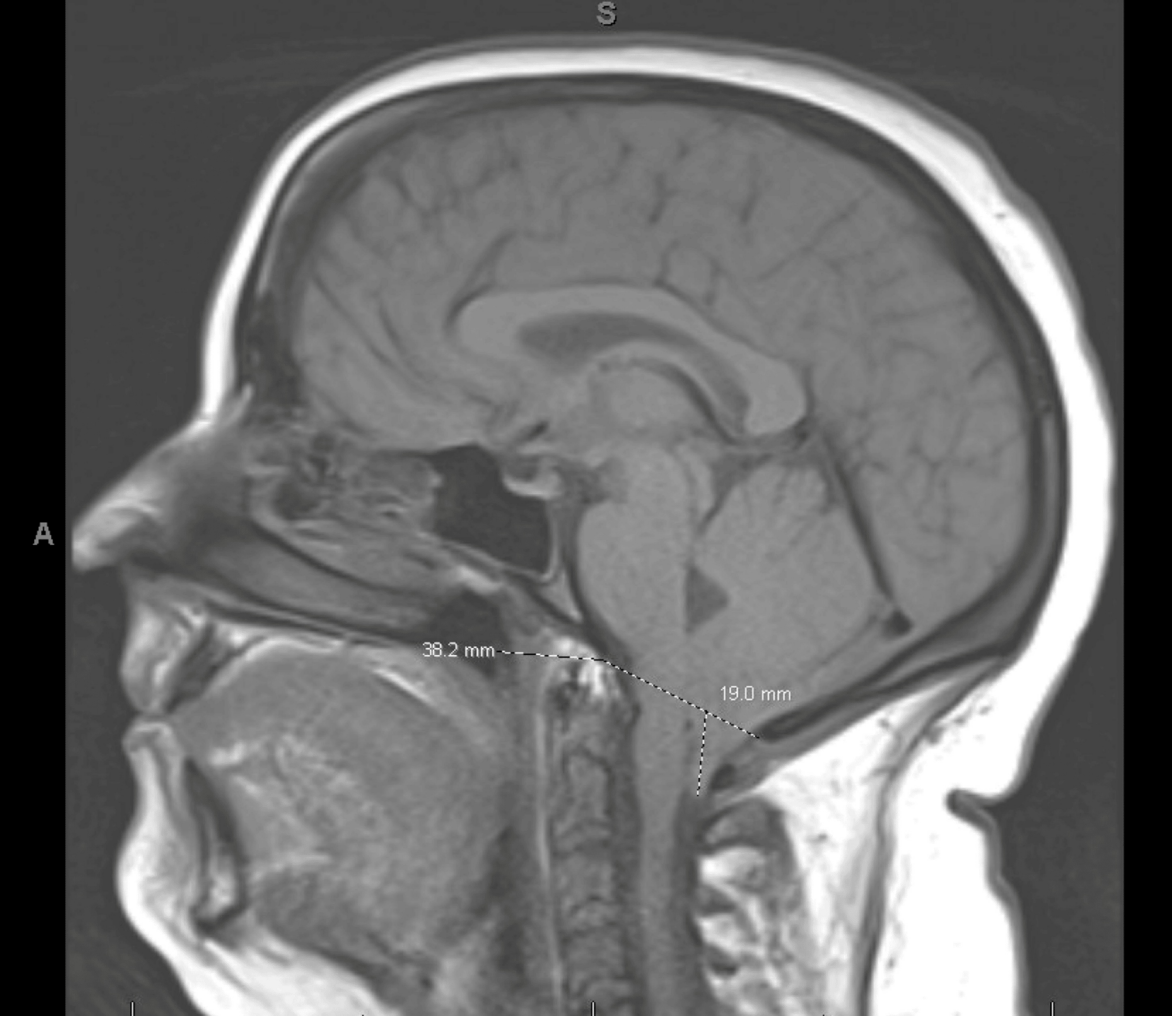
Progression of cerebellar tonsillar herniation, seen here at 19 mm

A 28-year-old woman with a recent diagnosis of AML (FLT D835 mutation with deletion in the long arm of chromosome 11), along with a history of migraines, was admitted for her third cycle of high-dose cytarabine (HIDAC) consolidation therapy. She was diagnosed with AML after presenting with right axilla pain and fever, with labs showing an elevated white blood cell count of 28,000/µL, hemoglobin of 8.8 g/dL, and low platelet count of 8,400/µL. Smear showed peripheral blasts, and bone marrow biopsy revealed myelodysplasia-related AML with complex karyotype on chromosomal analysis and presence of FLT3 D835 mutation on FLT3 and next-generation sequencing (NGS) analysis. She underwent 7 + 3 induction therapy with Midostaurin and followed by cycles of HIDAC. On the second day of cycle #3, she developed bifrontal and bitemporal headaches accompanied by photophobia, phonophobia, and nausea. This headache was similar in character to her prior headaches, other than the prolonged duration and increased severity, with the absence of meningeal signs, fever, chills, or seizure-like activity. Treatment for status migrainosus with a combination of diphenhydramine, ketorolac, and prochlorperazine, valproate, magnesium sulfate, IV fluid bolus, dihydroergotamine, and dexamethasone provided little relief. An MRI of the brain performed on day 2 revealed progression of the Chiari malformation, with tonsillar herniation increasing to 19 mm from 15 mm noted on an MRI taken two months prior, indicating significant descent below the foramen magnum, without any abnormal signal changes in the brainstem as shown in Figure 1.

**Figure 2:**
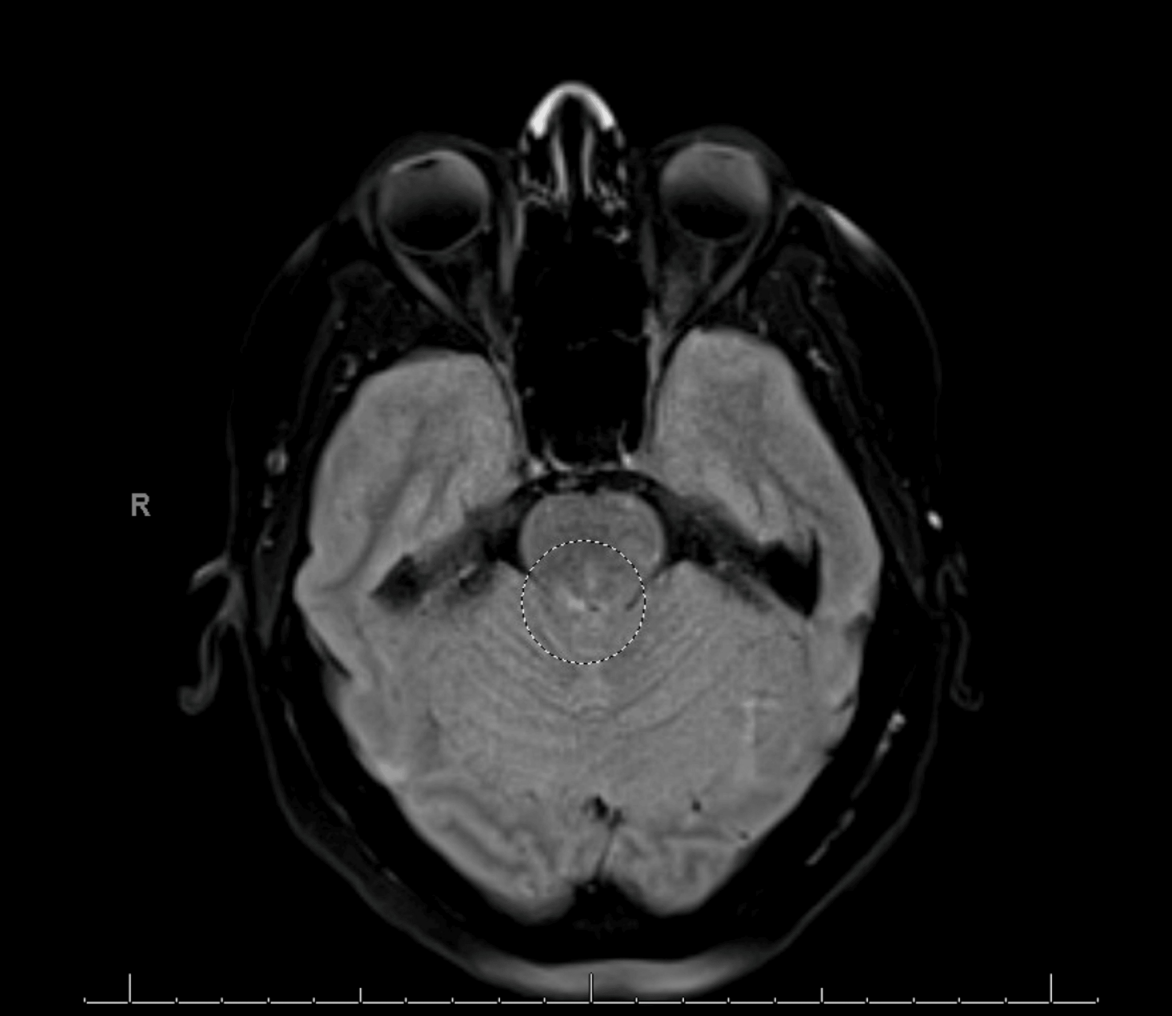
Mild increased FLAIR signal in the bilateral middle cerebellar peduncles without corresponding restricted diffusion seen on MRI brain WWO, showing brain section at the level of the pons FLAIR: fluid-attenuated inversion recovery, WWO: with and without.

**Figure 3:**
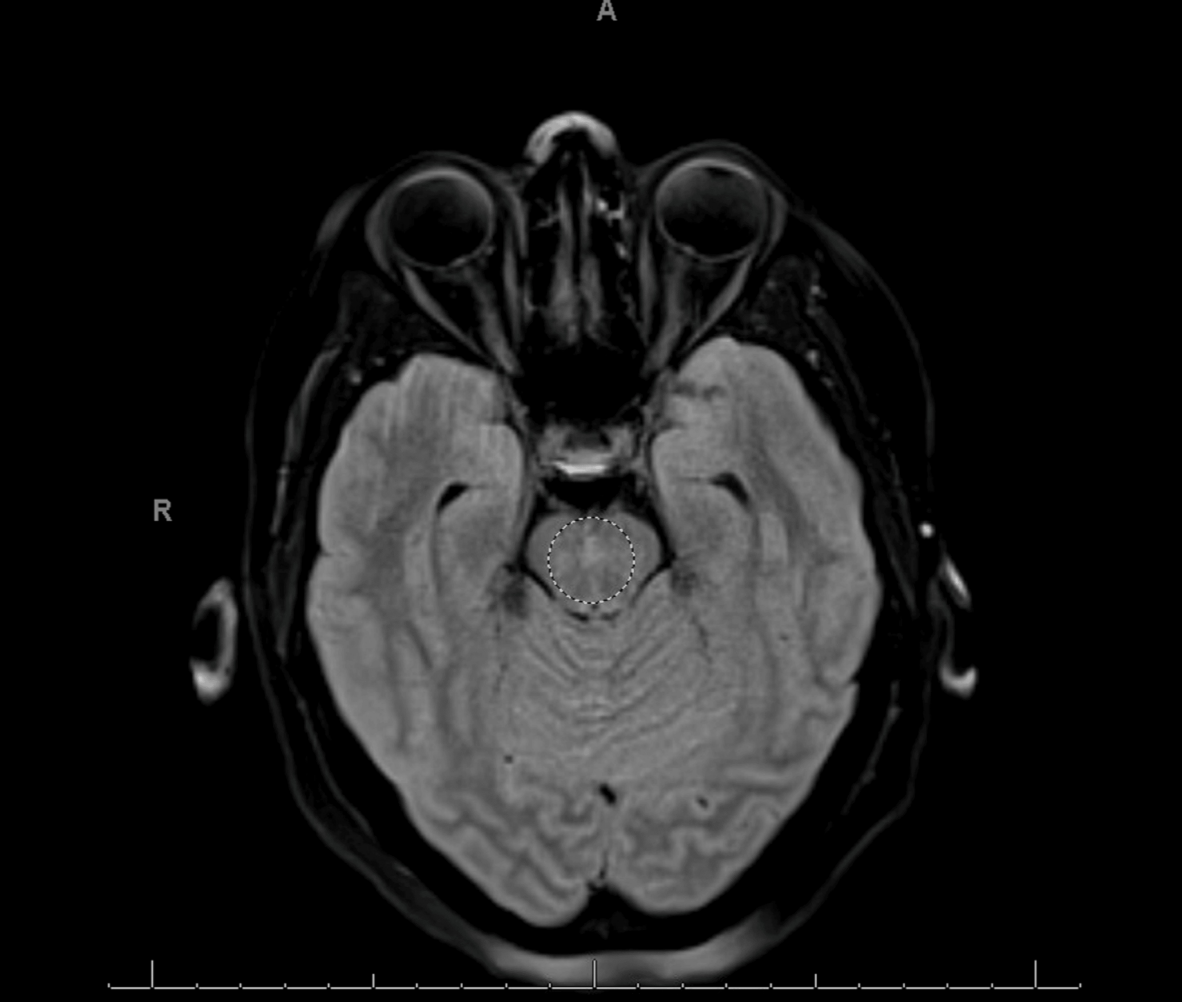
Mild increased FLAIR signal in the bilateral middle cerebellar peduncles without corresponding restricted diffusion seen on MRI brain WWO, showing brain section at the level of midbrain FLAIR: fluid-attenuated inversion recovery, WWO: with and without.

On day 4, the patient developed a bilateral (R>L) cranial nerve (CN) VI palsy and grade III papilledema. The brain MRI was repeated and now showed new, non-enhancing, patchy areas of increased fluid-attenuated inversion recovery (FLAIR) signal in the dorsal pons, as well as in the bilateral cerebral and middle cerebellar peduncles as shown in Figures 2, 3.

It is not common to routinely screen AML patients for CNS invasion to avoid the potential seeding of leukemia cells into the CSF in case of a traumatic lumbar puncture (LP). In our patient's case, obtaining LP was relatively contraindicated with worsening CM, posing risk of tonsillar herniation after LP due to increase in the pressure gradient between the intracranial and intraspinal compartment. On day 9, the patient underwent a suboccipital craniotomy, C1 laminectomy, and decompression of foramen magnum for concern of increased intracranial pressure due to tonsillar herniation. The patient was noted to have new partial right upper and lower facial weakness, double vision, and tongue weakness suggesting cranial nerve IV, VII, and XII palsy on the same day. MRI brain on day 9 showed increase in the hyperintensity in cerebral peduncles and inferior cerebellar peduncles. Following decompression, a lumbar puncture revealed increased opening pressure and a myeloid blast population comprising 98% of the cells, confirming CNS involvement by AML. Intrathecal chemotherapy planned to start 14 days after surgery for wound healing. Four days prior to initiating intrathecal chemotherapy, the patient experienced an episode of asystole of unclear cause. Cardiopulmonary resuscitation was performed but was unsuccessful.

## Discussion

Differentiating between Chiari I malformation and central nervous system (CNS) involvement by acute myeloid leukemia (AML) can pose a diagnostic challenge due to their similar presentation of headaches and cranial neuropathies. While CNS infiltration by AML is relatively uncommon at initial diagnosis, with incidence rates reported between 1% and 33% in adult patients, it remains a critical consideration given its implications for treatment and prognosis [1,2].

In this case, the initial MRI of the brain did not show signs suggestive of leukemic infiltration, which aligns with prior literature noting that radiographic evidence is often absent in early CNS-AML, making AML involvement a less likely early consideration [1,2]. This contributed to an initial diagnostic focus on alternative etiologies, including status migrainosus and progression of Chiari I malformation. Status migrainosus was initially high on the differential diagnosis because the patient’s headaches closely resembled their typical migraine pattern, likely triggered by the discontinuation of Erenumab two months prior to initiating chemotherapy for AML. Assessment for papilledema was further limited because a fundus examination could not be performed due to the patient’s sensitivity to bright light. Similar diagnostic dilemmas have been described in other reports where coexisting neurological conditions masked leukemic CNS involvement [3,4].

The progressive cranial nerve deficits, beginning with cranial nerve IV and extending to cranial nerves VII and XII, were key to revisiting the differential diagnosis. This pattern of multifocal cranial neuropathies is consistent with prior observations that CNS-AML can mimic inflammatory or compressive neuropathies before cytological confirmation [5,6]. The lumbar puncture following occipital craniotomy proved decisive, revealing elevated opening pressure and myeloid blasts comprising 98% of cerebrospinal fluid (CSF) cells. Similar findings have been emphasized by Del Principe et al., who recommended that CSF analysis be performed in all AML patients with new or progressive neurological signs, regardless of imaging results [7].

Compared to previously reported cases, this case highlights the challenges in early detection of CNS-AML, especially when coexisting neurological abnormalities such as Chiari malformation or history of migraines confound the clinical picture. While Ganzel et al. reported that CNS involvement at diagnosis did not significantly affect remission or survival outcomes, more recent analyses suggest that early identification and targeted intrathecal therapy may improve CNS disease control [2,8]. The presence of high-risk cytogenetic or molecular markers has also been associated with increased likelihood of CNS infiltration, reinforcing the need for vigilance even in the absence of typical radiographic findings [9,10].

Ultimately, this case underscores that a normal MRI does not rule out CNS leukemia and that clinical progression should guide timely lumbar puncture and cytological evaluation. Early diagnosis remains essential for optimizing management and preventing irreversible neurological deficits. Incorporating proactive CSF analysis into evaluation protocols for AML patients with neurological symptoms may help improve detection and outcomes.

## Conclusions

This case underscores the importance of vigilant screening for central nervous system (CNS) involvement in acute myeloid leukemia (AML) patients with high-risk mutations who present with neurological symptoms. Early recognition is crucial for timely diagnosis and appropriate management. While CNS involvement in AML is generally considered rare, reported incidence varies widely depending on the population studied and the diagnostic criteria applied. Some evidence suggests that the true rate may be higher if lumbar punctures are performed routinely in all patients. This highlights the need for active screening and a high index of suspicion, rather than relying solely on clinical presentation or brain imaging. Timely and accurate diagnosis is essential for guiding appropriate interventions and potentially improving patient outcomes.
